# Connectivity and nutrient enrichment affect the productivity and stability of aquatic meta-ecosystems

**DOI:** 10.1098/rspb.2025.1197

**Published:** 2025-11-12

**Authors:** Egor Katkov, Michelle Gros, Vincent Fugère, Aleksei Sychterz, Christina P. Tadiri, Rowan D. H. Barrett, Melania E. Cristescu, Gregor F. Fussmann, Andrew Gonzalez

**Affiliations:** ^1^ Department of Biology, McGill University, Montreal, Canada; ^2^ Quebec Centre for Biodiversity Science, Quebec, Canada; ^3^ Département des Sciences de l'environnement, Université du Québec à Trois-Rivières, Trois-Rivières, Quebec, Canada; ^4^ School of Natural Sciences, University of Galway, Galway, Ireland; ^5^ Great Lakes Institute for Environmental Research, University of Windsor, Windsor, Ontario, Canada

**Keywords:** meta-ecosystem, zooplankton, phytoplankton, mesocosm, nutrient enrichment, eutrophication, connectivity, freshwater

## Abstract

Despite major human impacts on aquatic connectivity (e.g. channelization, damming) and on nutrient inputs (e*.*g. agriculture, sewage), empirical studies on the combined impacts of these effects are rare. To better understand the interactive role of connectivity and nutrient enrichment in shaping meta-ecosystem stability, we set up a mesocosm experiment mimicking a minimal meta-ecosystem composed of two ponds (upstream and downstream). The upstream pond received varying water volumes from a mesotrophic lake (ranging from 0% to 40% mesocosm volume per week) and five levels of nutrient enrichment (phosphorus and nitrogen). The experiment featured a fractional factorial design, with 13 unique treatment combinations monitored over 14.5 weeks. We found that connectivity increased phytoplankton biomass in highly nutrient-enriched meta-ecosystems, that connectivity and nutrient enrichment independently promoted synchrony and spatial homogeneity of phytoplankton biomass within meta-ecosystems and that our treatments did not influence temporal stability beyond the initial nutrient-induced biomass increase. Furthermore, while intermediate levels of connectivity stimulated zooplankton biomass and diversity, the increase was counteracted with nutrient enrichment. We conclude that increased ecosystem connectivity is likely to exacerbate the negative effects of nutrient enrichment on freshwater meta-ecosystems across watersheds while homogenizing temporal population dynamics.

## 1. Introduction

Aquatic ecosystems and their communities form highly connected networks, with directional flows that mediate their diversity and productivity. However, the role of this connectivity in the context of nutrient-driven eutrophication [1] is not totally clear. Classical models suggest that in ecosystems with at least two trophic levels, nutrient enrichment can cause temporal instability, measured by the coefficient of variation (ratio of the standard deviation to the mean) of biomass or population size [2,3]. To understand how local instability might propagate through multiple connected aquatic ecosystems (i*.*e. a meta-ecosystem), McCann *et al*. [4] studied a mathematical model where ecosystems with two trophic levels were linked with directional flows of nutrients or individuals. McCann *et al*. [4] predicted that nutrient enrichment in the most upstream ecosystem inevitably results in nutrients becoming concentrated in the downstream ecosystem, leading to strong local temporal instability. McCann *et al*. [4] also predicted that slowing the rate of nutrient flow can have a stabilizing effect on the downstream ecosystem. More recently, Olson & Jones [5] developed a model to mechanistically explain the relationship between chlorophyll *a* and total phosphorus concentrations, and important parameters included water residence time and concentrations of inflowing phosphorus (P). However, empirical tests for the predictions of these studies remain very limited.

A major threat to ecosystem stability in many aquatic ecosystems is eutrophication [1]. Briefly, nutrient runoff from anthropogenic sources, such as agriculture or sewage, can cause algal blooms which destabilize the rest of the ecosystem, with consequences for humans relying on the water body. For example, algal blooms can be inedible and even toxic for species at higher trophic levels, such as freshwater zooplankton, which support fish populations [6]. Bacterial decomposition of algal bloom die-offs can deplete oxygen levels, potentially causing mortality events of higher trophic species. Classical resource-competition models with two trophic levels show that, counter-intuitively, nutrient enrichment causes predator–prey cycles to emerge [2].

Other forms of anthropogenic change, such as the channelization of waterways, dredging and other forms of land-use change, can increase the rate at which nutrients end up in distant ecosystems [7,8]. Several studies have found that local metrics of connectivity (the movement of organisms and the flow of nutrients), such as water residence times, increase aquatic ecosystem stability in a nutrient loading context [9,10]. However, such studies do not consider the effects that water residence times might have on downstream water bodies.

Meta-ecosystem theory predicts that the exchange of individuals of different species between spatially distinct communities (i*.*e*.* dispersal within meta-communities) will stabilize local populations and total biomass production in the presence of environmental fluctuations. This effect, termed ‘spatial insurance’ [11], is mediated by the number of ecosystems, the pattern of connections and the spatiotemporal correlation in the environment [11–15]. However, high rates of dispersal can also lead to synchrony among patches, which increases the risk of global species extinctions as the meta-community behaves more like a single large community [15–17]. As a result, low to intermediate levels of dispersal were expected to allow for asynchronous biomass dynamics among patches while still allowing for the maintenance of local and regional diversity, thereby maximizing meta-community stability via the spatial insurance effect [18]. Increasing inputs of a limiting nutrient across a metacommunity were found to enhance local and regional stability arising from spatial insurance effects by increasing species’ biomass production (productivity) and local and regional diversity [12]. The concept of spatial insurance can also be extended to meta-ecosystems [19], which consider ecosystem-level processes and flows of resources and energy between connected ecosystems [20]. An open question is the degree to which the rate of connectivity (flow or movement) among local ecosystems and the limiting factors driving ecosystem processes (e.g. nutrient enrichment) interact to mediate community and ecosystem stability.

In aquatic landscapes, planktonic organisms are carried by water downstream, together with nutrients. However, in most theoretical and empirical studies on meta-ecosystems, species dispersal is dissociated from nutrient fluxes and often lacks directionality [21]. McCann *et al*. [4] addressed the problem of directionality by modelling the movement of organisms and nutrients as directional diffusion, leading to resources and biomass to accumulate in the terminal node of the network. However, directional diffusion neglects the movement of the water that is used to transport the nutrients and biomass, and which prevents nutrients from becoming increasingly concentrated downstream. On the other hand, Olson & Jones [5] and Jones *et al*. [22] explicitly model water movement as residence times, or drainage ratios, but focus on the ecosystem scale. Tadiri *et al*. [23] used a laboratory gradostat experiment to show that connectivity can propagate, and in some cases, amplify the destabilizing effects of nutrient enrichment. These results highlight the need to test for the interactive effects of connectivity and nutrient enrichment in more natural systems.

To our knowledge, few empirical studies (e.g. [23]) have looked at the interaction between nutrient enrichment and connectivity on the dynamics or stability of aquatic meta-ecosystems. To test existing theory indicating an interactive role of connectivity and nutrient enrichment on meta-ecosystem biomass and stability [4], we set up a mesocosm experiment composed of two ponds connected by directional, weekly water transfers (upstream and downstream). The upstream ponds received different levels of nutrient enrichment (between 0 and 200 μg P l^−1^ per week, along with nitrogen, in ratios proportional to what was observed in the system) and were connected to the downstream ponds by transfers ranging from 0% to 40% of pond volume per week. Although meta-ecosystem models (e.g. [4]), gradostat experiments (e.g. [23]) and steady stream inflow (e.g. [5]) represent connectivity as continuous, weekly transfers can be thought of as storm-driven pulses, while also providing a good mathematical approximation for continuous flow (electronic supplementary material, figure S2) and simplifying experimental logistics. This design allowed us to detect and characterize any nonlinear effects of nutrient enrichment and connectivity that we could expect from meta-ecosystem theory (e.g. spatial insurance [11], or light limitation at low water residence times [5]).

At the outset, we hypothesized that nutrient enrichment and connectivity would have synergistic effects increasing phytoplankton biomass. Specifically, we expected that, when combined with nutrient enrichment, low levels of connectivity would cause nutrients and algal biomass to remain trapped in the upstream pond, resulting in limited total meta-ecosystem biomass. Conversely, the highest levels of connectivity would cause nutrients and algal biomass to leave the meta-ecosystem too rapidly to maximize biomass. Intermediate levels of connectivity were expected to maximize algal biomass at the meta-ecosystem scale by stimulating growth in the downstream pond while allowing the meta-ecosystem to retain most nutrients. Second, we hypothesized that high rates of connectivity, especially when paired with nutrient enrichment, would generate synchronized dynamics (with some temporal lag between the upstream and downstream ponds). In other words, we expected nutrients to stimulate growth in upstream ponds, which, with high connectivity, would also occur in the downstream pond. Third, we hypothesized that the temporal coefficient of variation (CV=σ/μ, where σ and μ are, respectively, the standard deviation and mean of a time-series of some measure of population size [24,25]) of phytoplankton biomass would increase with nutrient enrichment, for example, due to oscillatory consumer-resource dynamics. However, we also hypothesized that this nutrient-induced decrease in stability should be attenuated by intermediate levels of connectivity, as per the spatial insurance hypothesis [11]. Finally, we hypothesized that the stability of the zooplankton community, as measured by its biomass, and Hill diversity [26] would be negatively impacted by nutrient enrichment but stimulated by intermediate rates of connectivity as per the spatial insurance hypothesis [11].

## 2. Methods

### (a) Experimental setup and design

Our experiment was conducted between the months of July and October 2022 at the Large Experimental Array of Ponds (LEAP) facility, located at the Gault Nature Reserve in Mont-Saint-Hilaire, QC, Canada. The LEAP is an open gravel-covered area (1300 m^2^) within a 1000-hectare protected forest, equipped with an intake reservoir and a retention tank for discharge water. Unfiltered lake water is provided through a drainage pipe directly from Lac Hertel (45°32′ N, 73°09′ W), a headwater lake, and is stored in a 100 000 l intake reservoir. With a gravity-powered underground plumbing system, water from the reservoir was used to fill 1136 l stock tanks (Rubbermaid), referred to herein as ponds. These ponds were open to the atmosphere and were thus exposed to evaporation and precipitation, both of which were relatively balanced to within ±5% of the initial filling volume over the course of the experiment. Prior to filling, the ponds were scrubbed clean with tap water. After allowing for a 1-week stabilization period, chlorophyll *a* and nutrient concentrations were measured to account for variability prior to treatment application. Treatments then commenced on 4 July 2022, marking the start of the 15-week experiment, which ended on 13 October 2022.

Our study design paired two ponds together, forming two-patch meta-ecosystems. An exception was made for meta-ecosystems with no connectivity, where the meta-ecosystems consisted of single ponds, which we consider to be upstream for the purposes of the nutrient treatment and subsequent analyses. Although we avoided re-using data for model fitting and statistical tests, ponds with zero nutrient enrichment and zero connectivity can also be seen as downstream ponds in meta-ecosystems with any level of nutrient enrichment but zero connectivity and were used as such for calculating meta-ecosystem-level statistics such as cross-correlation and CVs. Each meta-ecosystem had one of five rates of connectivity and nutrient additions, with 13 unique treatment combinations (electronic supplementary material, figure S1). A set percentage of water (0%, 10%, 20%, 30% or 40% of the total pond volume) was displaced once a week from the upstream to the downstream pond using 10 l graduated buckets. First, water was removed from the downstream pond and discarded to make room for the transfer of water from the upstream pond. Finally, the upstream pond was refilled using water from the reservoir (electronic supplementary material, figure S1). The 0% week^−1^ ponds were treated as isolated systems where no water entered or exited. To ensure even turbulence across all treatments, ponds with less than 40% connectivity had buckets of water removed and immediately added back into the same pond to mimic the turbulence experienced by the 40% week^−1^ treatment. Independently of the connectivity treatment, the nutrient treatment was applied by weekly additions of two forms of phosphorus (KH_2_PO_4_ and K_2_HPO_4_) and nitrogen (KNO_3_) to upstream ponds, while maintaining a previously established lake N : P molar ratio of 31 : 1 [27] (electronic supplementary material, figure S1). Nutrient spikes were added to the upstream pond of all meta-ecosystems after applying the connectivity treatments on the same day. A total of 46 ponds were filled and sampled in this design, including duplicates for each nutrient and flow rate combination depicted in electronic supplementary material, figure S1. In addition, the reservoir was sampled as a reference pond.

### (b) Sampling and laboratory analyses

Four times over the course of the experiment (30 June, 13 July, 30 August and 12 October), integrated water samples were collected for nutrient analysis. Phosphorus and nitrogen concentrations were assessed in both their total and dissolved forms by collecting raw water and water filtered through a 0.45 μm syringe filter, respectively. Nutrient samples were processed by the Groupe de Recherche Inter-universitaire en Limnologie (GRIL) laboratory at the Université du Québec à Montréal (UQAM) using standard protocols (see electronic supplementary material, Methods).

We sampled for phytoplankton twice a week: 3 and 7 days following weekly connectivity and nutrient treatments. Sampling was performed before treatment applications when they occurred on the same day. A PVC pipe (1 inch diameter) was used to collect integrated water samples of 1 l from the top 50 cm of each pond. Water was stored in dark bottles and left to sit in a dark room for at least 30 min and up to a maximum of 6 h at ambient temperature before being processed with a benchtop Fluoroprobe (bbe Moldaenke, GmbH). For each sample, the Fluoroprobe recorded 10 measurements, indicating the amount of total chlorophyll *a* (μg l^−1^) composed of four different algal groups based on pigment fluorescence: green algae (chlorophytes), blue-green algae (cyanobacteria), brown algae (diatoms and dinoflagellates) and cryptophytes. Tap water was used to rinse all equipment in between every sample. Additionally, in all 46 ponds (and the reservoir), water temperature, dissolved oxygen, pH and conductivity were monitored weekly using a multiparameter metre (YSI, ProQuatro).

Crustacean zooplankton were also collected five times over the course of the study by inserting an inverted 1 l Nalgene bottle into each pond and filling it by slowly tilting the bottle upright underwater. Water from the bottle was then sieved using a 30 μm mesh. This process was repeated until a total of 4 l of water was sieved. Collected samples on the mesh were submerged in carbonated water and 90% ethanol to fix the sample. Zooplankton samples were then rinsed into a sample cup and preserved in a 70% ethanol solution until analysis under a light microscope (Nikon SMZ800) by two counters. The entirety of each sample was counted, amounting to 4 l of pond water. Each treatment duplicate was counted by a set person, consistently at each time point. To correct for the counter’s biases, one pond was selected as a calibration pond and was counted by both individuals. Cladocera individuals were identified at least at the family level, grouping together *Bosmina* spp. and *Chydoridae* spp. In addition, copepods were grouped into a *Cyclopidae* group. In most cases, however, individual Cladocera were identified at the species level (electronic supplementary material, table S1).

### (c) Statistical analyses

All analyses were conducted using R v. 4.5.1. All plots were generated using the ggplot2 R package v. 3.5.2 [28]. To prevent overfitting of GAMMs, the (marginal) basis dimensions for connectivity and nutrient enrichment were always set to a maximum of 3.

Fluoroprobe readings from the same water sample were first averaged to obtain a single measurement per pond per sampling occasion. Samples with chlorophyll concentrations greater than 580 μg l⁻¹ were adjusted to account for self-shading of fluorescence, following the relationship depicted in electronic supplementary material, figure S3. To explain the temporal variation in the nutrient and phytoplankton population dynamics of different treatments, a generalized additive mixed model (GAMM) with log-transformed chlorophyll *a* as the dependent variable; pond position (upstream/downstream) as a fixed factor; connectivity, day and nutrient enrichment as interactive tensor products; and the pond identifier as a random factor was fitted using the mgcv R package v. 1.9.1 [29].

The coefficient of cross-correlation between the chlorophyll *a* concentrations of connected upstream and downstream ponds was calculated using the stats R package v. 4.5.1 [30]. First, a linear model with connectivity, nutrient enrichment and time lag as interacting categorical factors and cross-correlation as the numerical response was used to estimate a mean standard error across all treatment and lag values. The standard error was used to estimate a 95% confidence interval, which was Bonferroni-corrected for multiple comparisons across the 13 treatment combinations of the experiment. This 95% confidence interval was centred on a cross-correlation coefficient of zero and was used as a threshold to determine whether there was significant synchrony between connected ponds or not. Furthermore, the coefficient of cross-correlation was then used as the dependent variable in a GAMM with connectivity, nutrient enrichment, and different time lag values as interactive tensor products and the meta-ecosystem as a random slope using mgcv.

To calculate stability metrics for the meta-ecosystem, the average chlorophyll *a* value of the upstream and downstream ponds was taken. Next, two different estimators of CV were used: an unbiased estimator for low or moderately sized samples,


(2.1) $${\hat{c}}_{v}=\left(1+\frac{1}{4n}\right)\frac{s}{\overset{´}{x}},$$

where s is the sample standard deviation, n is the sample size and $\overset{´}{x}$ is the sample mean; and the geometric CV,


(2.2) $${\hat{c}}_{g}=exp{\left(s_{ln}^{2}-1\right)}^{1/2},$$

where $s_{ln}$ is the standard deviation of the natural log transform of the sample values. Whereas the small-sample-size CV estimator assumes a normal distribution for the data ($\hat{c_{v}}$), the geometric CV ($\hat{c_{g}}$) assumes a log-normal distribution [31], as is often the case for biomass data. The effects of treatments on both metrics were evaluated using a generalized additive model [29].

Zooplankton counts were converted to biomass using individual dry weight estimates from the literature [32]. Hill diversity was calculated using the vegan R package v. 2.6.10 [33]. GAMMs were used to test the effects of connectivity and nutrient enrichment on zooplankton biomass and Hill numbers as for chlorophyll *a*. To account for undetectable levels of zooplankton biomass in some ponds, a censored normal family with a log-link function was used.

## 3. Results

### (a) Nutrient treatment

**Figure 1.**
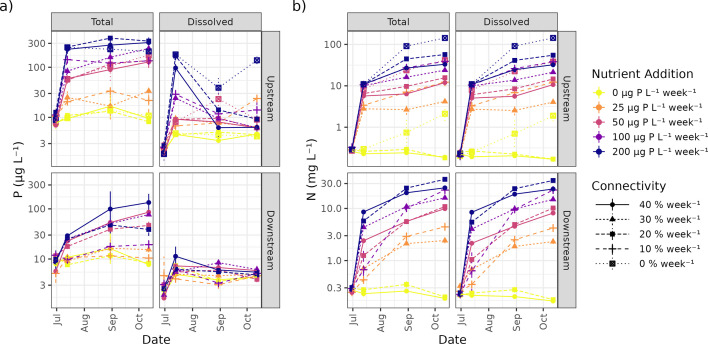
The total and dissolved fractions for (a) phosphorus and (b) nitrogen across time points. The effect of nutrient enrichment (colour) and connectivity (point shape and line type) on nutrient concentrations (*y*-axis) is shown for the upstream and downstream ponds.

Nutrients were most concentrated in the upstream ponds, where they were added weekly, compared to the downstream ponds (figure 1; in (b), note the difference in *y*-axis scales between the top and bottom panels). In meta-ecosystems with nutrient enrichment, over time, connectivity increased total phosphorus concentrations in downstream ponds but did not measurably affect upstream ponds (figure 1a; electronic supplementary material, table S2, figure S4a). Also, in meta-ecosystems with nutrient enrichment, over time, connectivity resulted in lower dissolved phosphorus concentrations in upstream ponds but had little to no effect in downstream ponds (figure 1a; electronic supplementary material, table S3, figure S4b). In other words, dissolved phosphorus was rapidly taken up by organisms by the time it arrived in downstream ponds, but was sometimes present in excess in some of the upstream ponds, especially those with high nutrient enrichment and low connectivity. Total and dissolved nitrogen concentrations both held similar values at comparable times and treatments (figure 1b), suggesting that most of the added nitrogen remained in the dissolved state instead of being taken up by the phytoplankton. In upstream ponds, nitrogen concentrations increased more slowly in more strongly connected upstream ponds (figure 1b; electronic supplementary material, tables S4 and S5, figure S4c,d). In downstream ponds, nitrogen concentrations were higher by the second week of the experiment in more strongly connected downstream ponds but equilibrated to similar values regardless of connectivity by the eighth week of the experiment (figure 1b; electronic supplementary material, tables S4 and S5, figure S4c,d). Together, these results suggest that phosphorus was more strongly limiting than nitrogen, particularly in downstream ponds, because nitrogen was added in great excess and behaves like an inert tracer.

### (b) Chlorophyll *a* response

**Figure 2.**
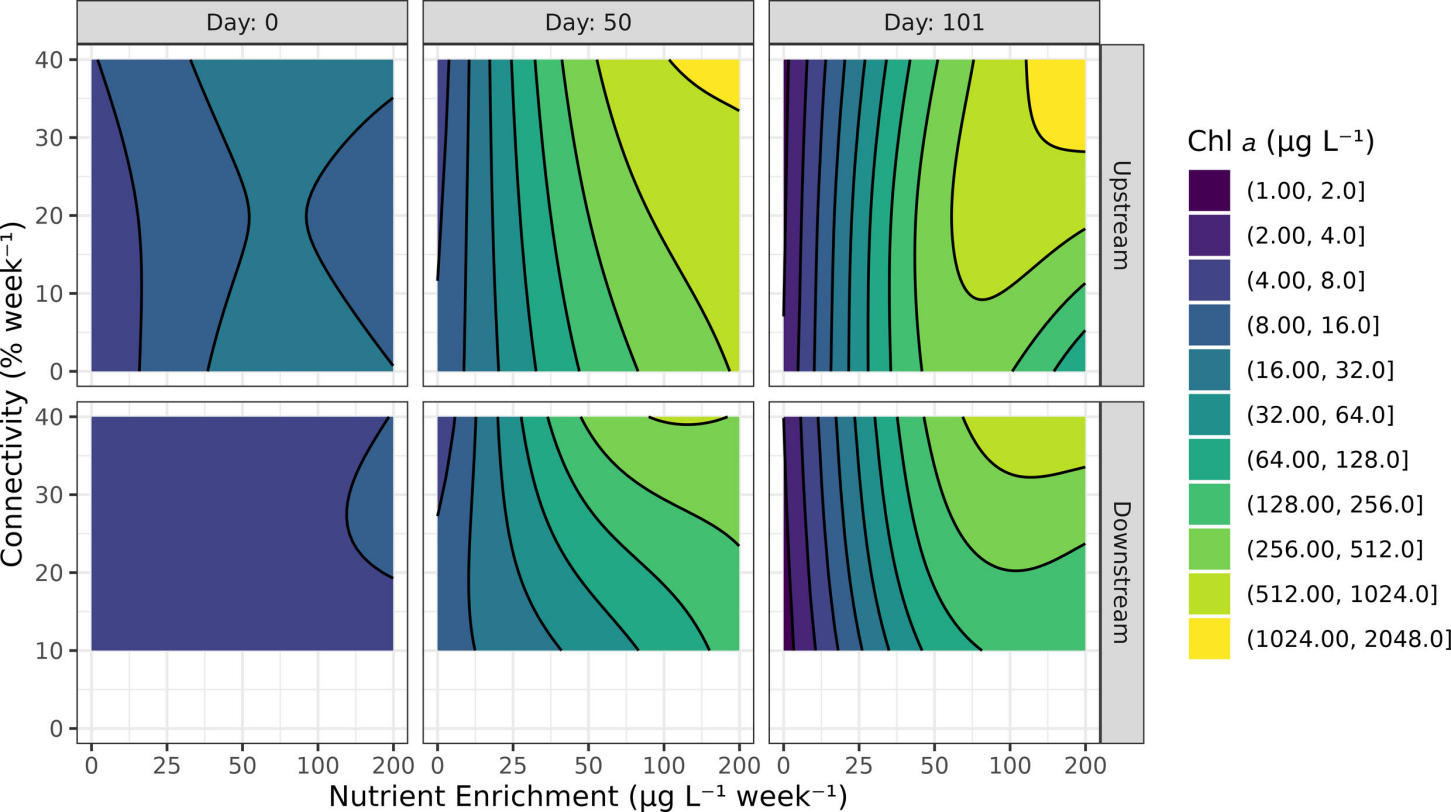
GAMM-modelled chlorophyll *a* biomass response (colour, contour lines) to nutrient enrichment (*x*-axis) and connectivity (*y*-axis) at select time points of the experiment in upstream and downstream ponds. See electronic supplementary material, figure S6, for underlying data.

Overall, we found that the GAMM explained 89% of the null deviance in the log chlorophyll *a* response (a proxy for phytoplankton biomass). We found a significant interactive effect of connectivity, nutrient enrichment, and time on the chlorophyll *a* concentration in both upstream and downstream ponds (figure 2; electronic supplementary material, table S6). In upstream ponds with nutrient enrichment of at least 25 µg P l⁻¹ week⁻¹, biomass was generally higher in more connected meta-ecosystems with 20, 30 or 40% week^−1^ connectivity than in ponds with 0 or 10% week^−1^ connectivity, especially towards the end of the experiment (figure 2, top row; electronic supplementary material, figure S5). For example, in the upstream ponds on the final day (101) of the experiment, in the 200 µg P l⁻¹ week⁻¹ treatment, the GAMM-modelled chlorophyll *a* concentrations were 8.8 (95% CI: [4.3, 18]) times greater in the 20% week^−1^ than the 0% week^−1^ connectivity ponds, and an additional 3 (95% CI: [1.5, 6.1]) times greater in the 40% week^−1^ relative to the 20% week^−1^ ponds. In the connected downstream ponds (same conditions as previous sentence), chlorophyll *a* concentrations were 4.1 (95% CI: [1.35, 12.6]) times greater in the 40% week^−1^ versus the 20% week^−1^ ponds. On the other hand, in the 0 µg P l⁻¹ week⁻¹ treatment, no significant differences in chlorophyll *a* biomass were found in neither upstream nor downstream ponds at any time point. This latter finding suggests that the connectivity treatment was not a significant disturbance or mortality event for the phytoplankton community.

In meta-ecosystems with nutrient enrichment, even the lowest level of connectivity (10% week^−1^) was sufficient to generate increased phytoplankton biomass in downstream ponds when compared to the no connectivity, no enrichment ponds (figure 2, in the bottom row, chlorophyll *a* increases with nutrient enrichment; electronic supplementary material, figure S6 bottom row compared to top-left facet). Contrary to our expectation, even the highest rates of connectivity were insufficient to cause a significant reduction in phytoplankton biomass in either upstream or downstream ponds (figure 2; electronic supplementary material, figure S5). At the scale of the meta-ecosystem, this means that when paired with nutrient enrichment, higher levels of connectivity generate greater levels of phytoplankton biomass and a greater amount of chlorophyll per gram of added phosphorus, suggesting better resource utilization by the algal community (electronic supplementary material, figure S7).

### (c) Cross-correlation analysis

**Figure 3.**
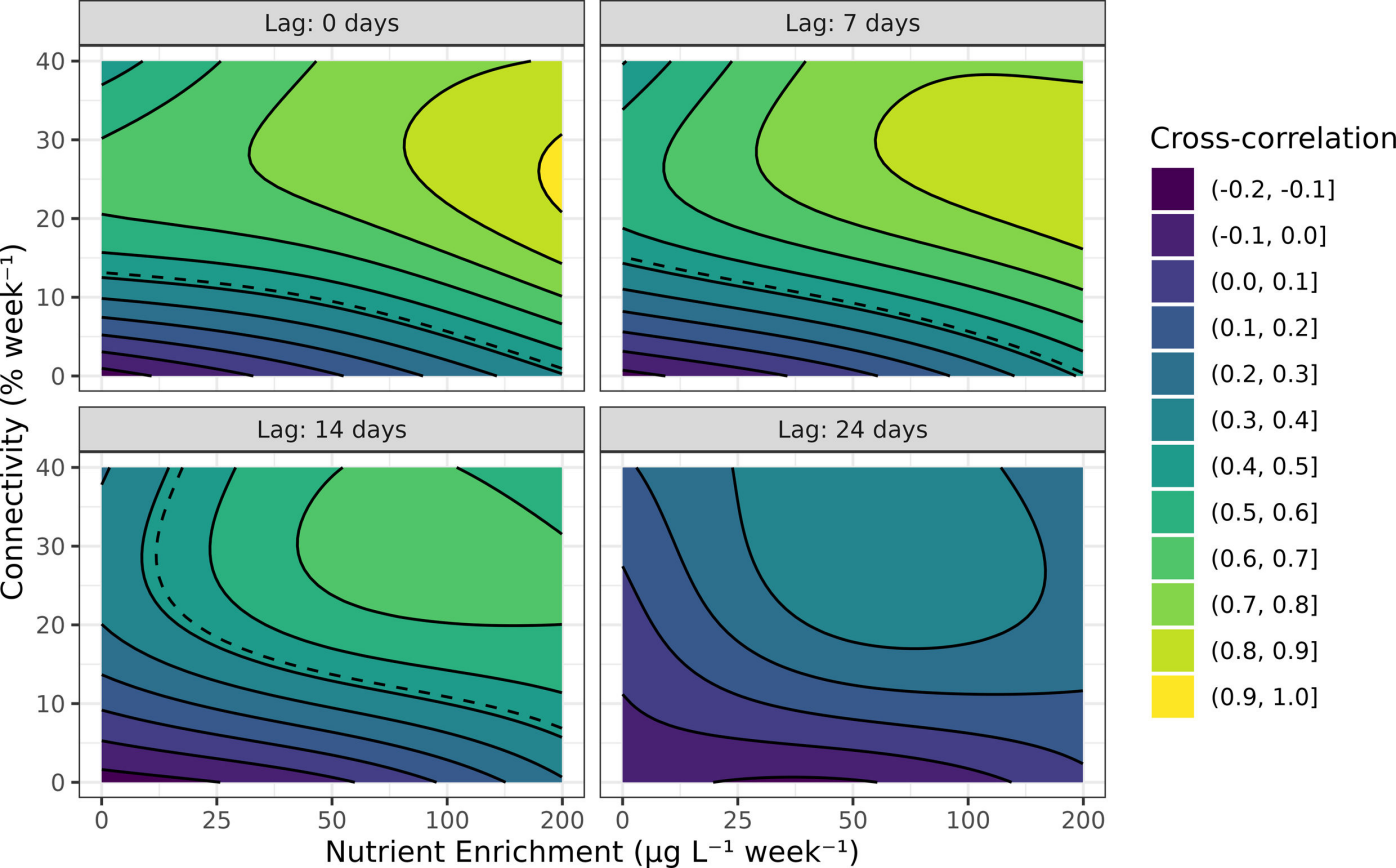
The modelled GAMM response of the cross-correlation coefficient between chlorophyll *a* in the upstream and downstream ponds (colour, contour lines) to nutrient enrichment (*x*-axis) and connectivity (*y*-axis) at different lag times. The dotted lines indicate an estimated threshold for values that are significantly different from zero, at the α=0.05 significance level, Bonferroni-corrected for all 13 treatment combinations. For the zero connectivity treatment, the ‘downstream’ pond was taken as one of the zero connectivity, zero enrichment ponds (that was not also the upstream pond). See electronic supplementary material, figure S8, for underlying data.

We found that the GAMM explained 62% of the null deviance of the cross-correlation coefficient between chlorophyll *a* in the upstream and downstream ponds response. The cross-correlation was controlled by (a) the interaction of lag time and connectivity and (b) the interaction of lag time and nutrient enrichment (electronic supplementary material, table S7). Both connectivity and nutrient enrichment had positive, but saturating effects on the cross-correlation coefficient (figure 3). The effect of both connectivity and nutrient enrichment was maximized at lag times of zero and decreased as the (absolute) lag time increased (figure 3). We estimated that at lag times under 10 days, the effect of connectivity alone can generate correlated (synchronous) dynamics at zero to low nutrient enrichment treatments (figure 3, top-left panel). At lag times of over 10 days, however, both nutrient enrichment and connectivity are required for synchronous dynamics between ponds to occur (figure 3).

### (d) Temporal stability

**Figure 4.**
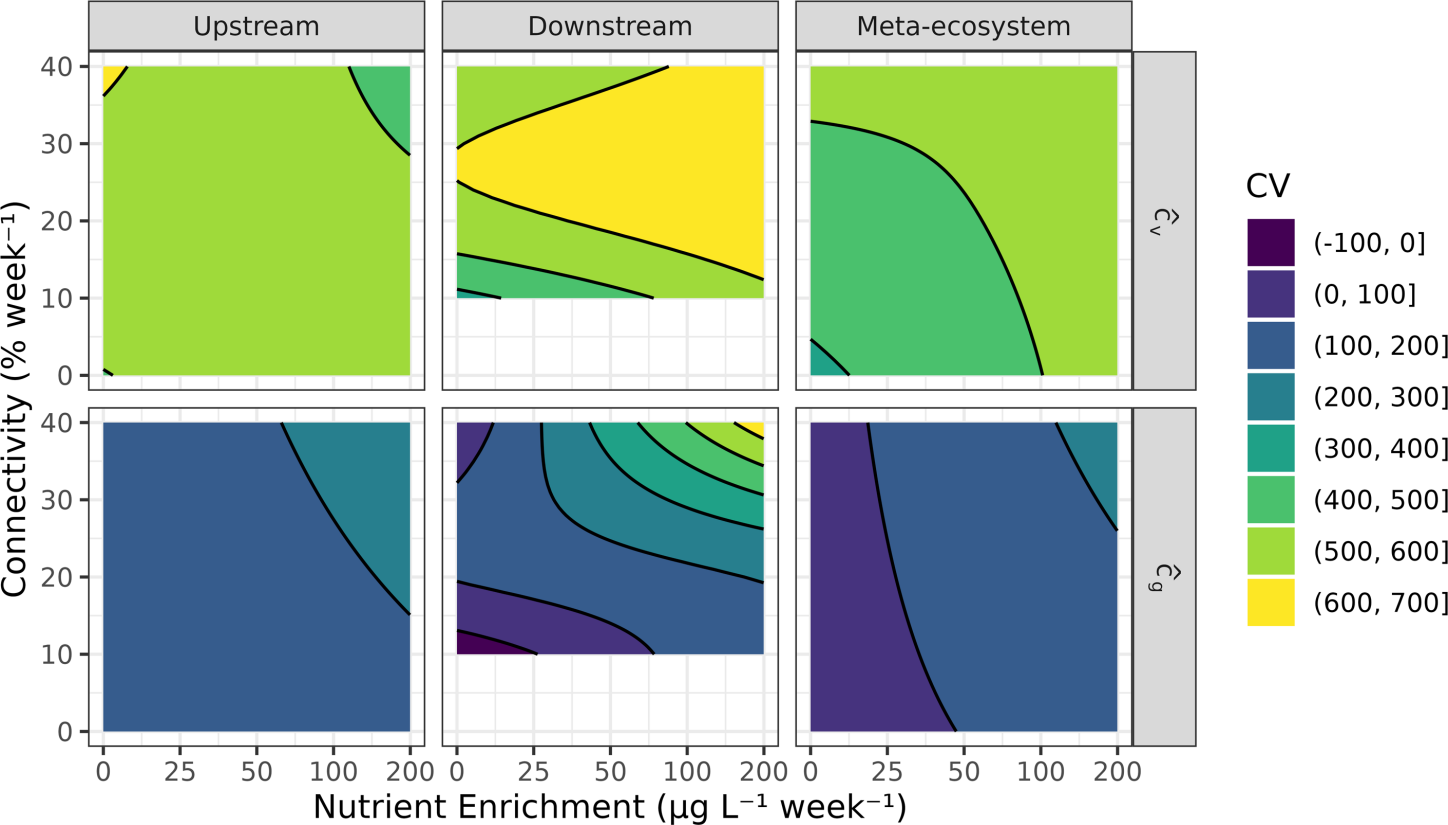
The modelled GAMM response of the CV of chlorophyll *a* over time (colour, contour lines) to nutrient enrichment (*x*-axis) and connectivity (*y*-axis). CVs were estimated for upstream ponds and downstream ponds, and at the meta-ecosystem level, using two different estimators: small-sample-corrected CV ($\hat{c_{v}}$; equation (2.1)) in the top row and geometric CV ($\hat{c_{g}}$; equation (2.2)) in the bottom row. See electronic supplementary material, figure S10, for underlying data.

We also found that different estimators for temporal variation in chlorophyll *a* (temporal CV) suggested different responses of biomass stability to our treatments. The more commonly used (small-sample-corrected) estimator for CV ($\hat{c_{v}}$; equation (2.1)) did not exhibit significant responses to any of the treatments in upstream or downstream ponds, or at the meta-ecosystem scale (figure 4; electronic supplementary material, tables S8 and S9). The geometric CV, on the other hand, increased in response to nutrient enrichment in the upstream ponds, downstream ponds, and at the meta-ecosystem scale as well as to connectivity in the downstream ponds and at the meta-ecosystem scale (figure 4; electronic supplementary material, tables S10 and S11). However, our results suggest that these responses are a consequence of the rapid, nutrient-induced growth in the first weeks of the experiment (figure 1; electronic supplementary material, figure S9, tables S12 and S13). Thus, although the addition of nutrients had a strong destabilizing effect at first, most ponds eventually reached a steady state as no oscillatory dynamics were observed. Additionally, Tukey-corrected pairwise contrasts revealed that there are no significant differences between any two levels of connectivity, despite the significant smooth term in the model, suggesting that the magnitude of the effect is very small (electronic supplementary material, table S14).

### (e) Zooplankton response

**Figure 5.**
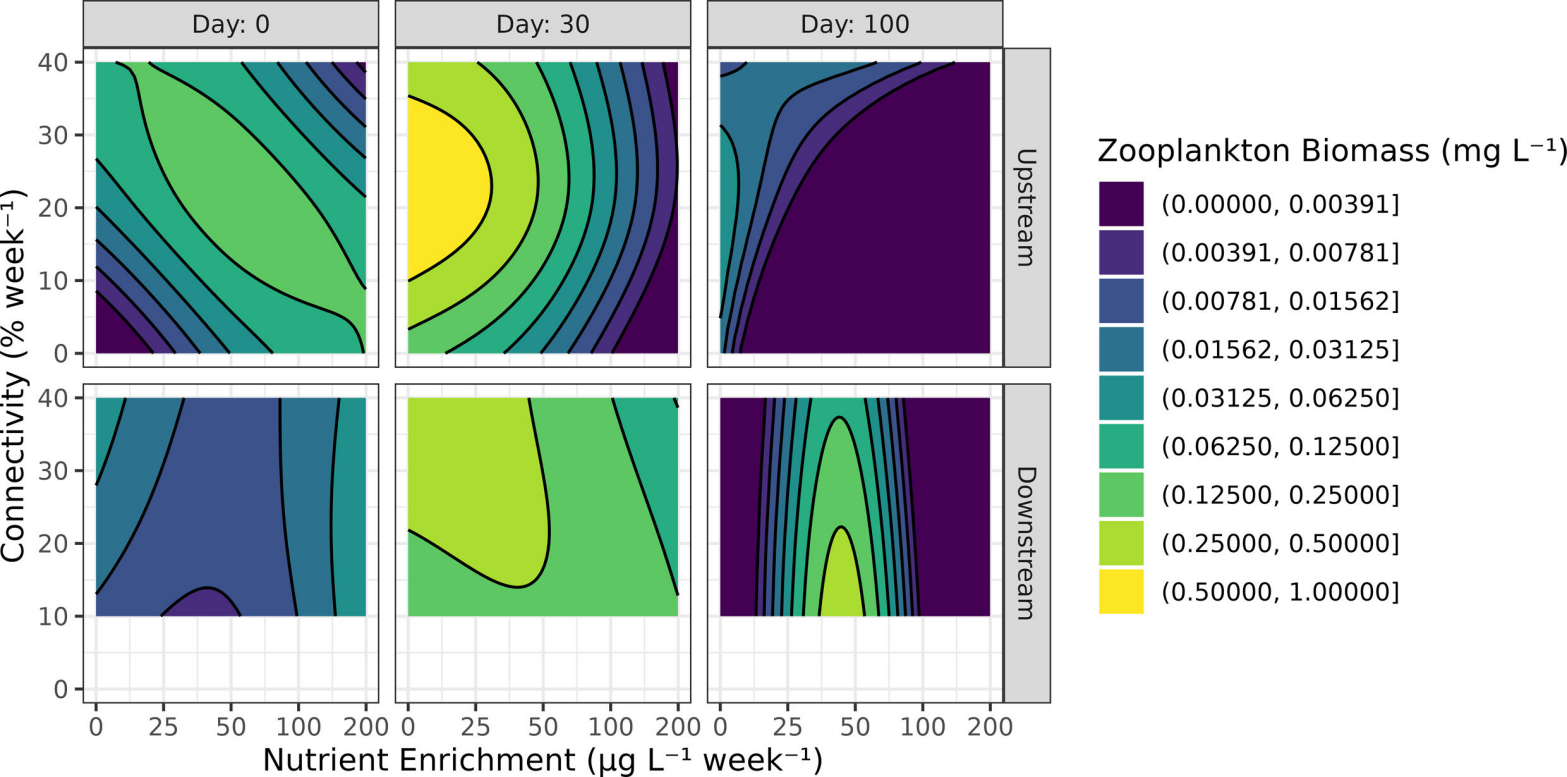
Zooplankton biomass response to experimental treatments. See figure 2 caption for details, and electronic supplementary material, figure S12, for underlying data.

Although we found that, on average, zooplankton biomass in the ponds was comparable to that of the reservoir, there was much more temporal variation in the ponds (electronic supplementary material, figure S12). Certain ponds experienced a strong zooplankton peak approximately 30 days into the experiment, followed by a crash of variable intensity. By using a censored model, we were able to extract information on zooplankton biomass from the entire duration of the experiment and model the intensity of both peak and crash. Indeed, the censored GAMM model explained 91% of the null deviance for the zooplankton biomass response. In upstream ponds, zooplankton biomass was significantly affected by the interaction of connectivity and nutrients over time (electronic supplementary material, table S15). In the first half of the experiment, greater than average zooplankton biomass was observed at intermediate levels of connectivity and low levels of nutrient enrichment (figure 5, top row, centre column). Additionally, nutrient enrichment had a significant negative effect on zooplankton biomass in upstream ponds. By the end of the experiment, zooplankton presence in upstream was restricted to low nutrient and high-connectivity ponds (figure 5, top row, right column). In downstream ponds, zooplankton biomass was affected independently by two interactions: (a) nutrient enrichment over time and (b) connectivity over time. There was a positive effect of intermediate nutrient concentrations on zooplankton biomass on the last day of the experiment in downstream ponds. Also, on the last day of the experiment, a negative effect of connectivity on zooplankton biomass was observed in the downstream ponds.

**Figure 6.**
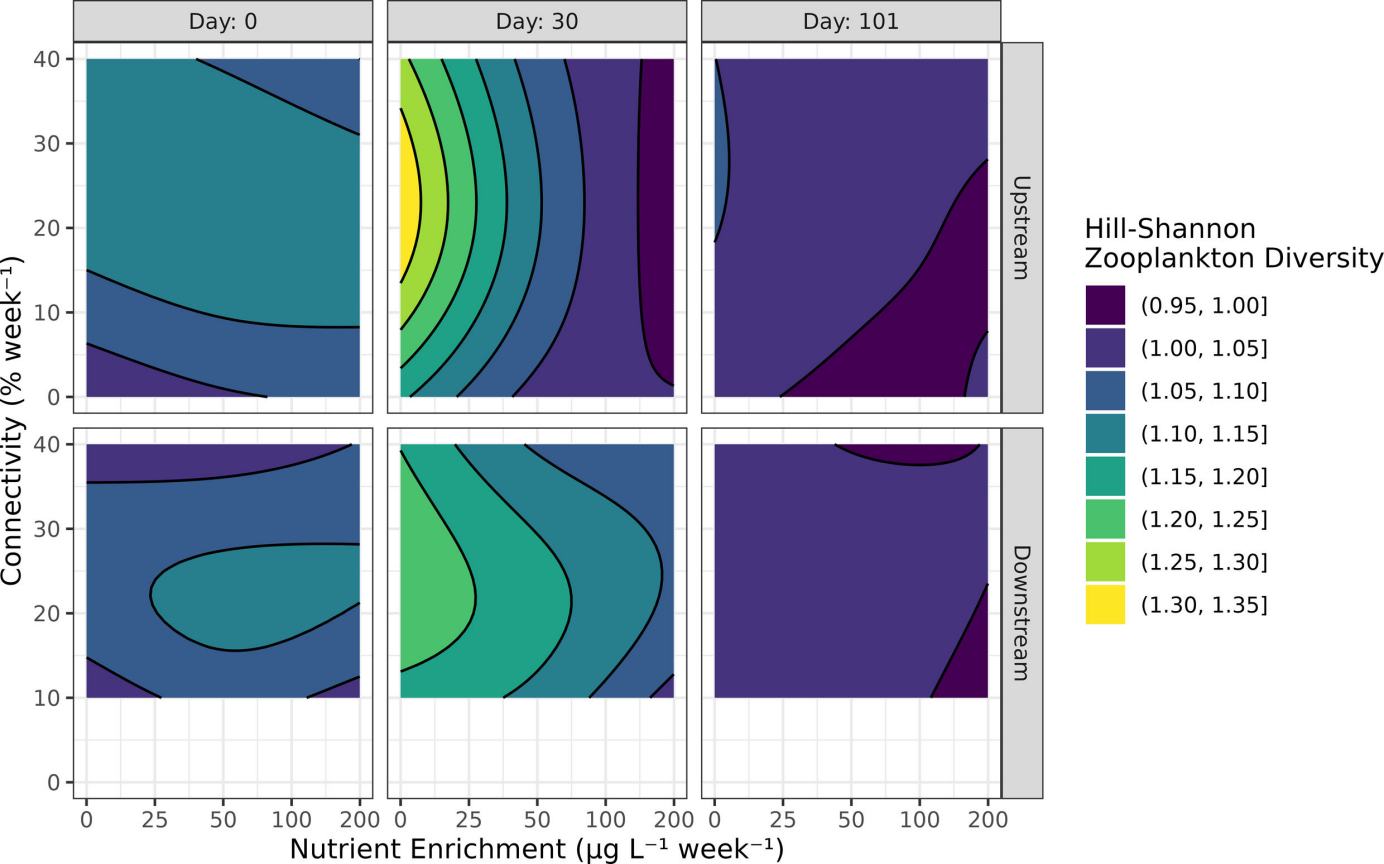
Hill–Shannon zooplankton diversity response to experimental treatments. See figure 2 caption for details, and electronic supplementary material, figure S15, for underlying data.

The fitted GAMM explained 67% of the null deviance of the Hill–Shannon diversity response. Hill–Shannon diversity was controlled by two interactions in upstream ponds: (a) connectivity and nutrient enrichment and (b) nutrient enrichment and time (electronic supplementary material, table S16). At low levels of nutrient enrichment, intermediate levels of connectivity maximized Hill–Shannon diversity in upstream ponds. However, increasing levels of nutrient neutralized any positive effects of connectivity in upstream ponds (figure 6, top row). Again, in the upstream ponds, Hill–Shannon diversity was maximized at low rates of nutrient enrichment within the first month after the start of the experiment. Over time, however, the positive effect of low rates of nutrient on Hill–Shannon diversity decreased in upstream ponds (figure 6, top row). In downstream ponds, Hill–Shannon diversity was similarly affected by (a) connectivity alone and (b) the effect of nutrient enrichment over time (electronic supplementary material, table S16). Intermediate levels of connectivity also maximized Hill–Shannon diversity in upstream ponds, but unlike in upstream ponds, nutrient enrichment did not have a negative interacting effect in downstream ponds (figure 6, bottom row). Similar to the upstream ponds, Hill–Shannon diversity was negatively affected by nutrient enrichment, an effect that disappeared at the end of the experiment.

Analysis of Hill–Simpson diversity (63% null deviance explained) and Hill-species richness (72% null deviance explained) mirrors those reported here for Hill–Shannon diversity (electronic supplementary material, figures S13 and S14). Species richness, however, was less sensitive than Hill–Simpson and Hill–Shannon diversities, and none of the described effect of connectivity was significant in the analysis of variance table (electronic supplementary material, tables S17 and S18).

## 4. Discussion

We investigated the role of connectivity in nutrient-enriched, semi-natural aquatic meta-ecosystems on the synchrony and stability of biomass production. Our two-pond experimental meta-ecosystems provided evidence of the interactive effects of nutrient enrichment and connectivity over time on phytoplankton biomass at the scale of individual ponds and of the meta-ecosystem, with greater overall biomass production in connected meta-ecosystems. Additionally, we showed that phytoplankton dynamics can become synchronized in connected ponds and that biomass fluctuations are more correlated across patches of more connected meta-ecosystems. However, we did not observe oscillatory dynamics in the phytoplankton biomass, with the nutrient-enriched ponds quickly reaching a new, high-biomass, temporally stable equilibrium which was unaffected by the dispersal treatment. We interpret our results in light of the spatial stability models that influenced the design of our experiment and make suggestions for future theoretical and experimental work [4,11,12].

Although the nutrient treatments were overall effective, we found that some of the added phosphorus was not accounted for during nutrient sampling. This may have been a result of uptake by periphyton, though we did not measure it [34]. Furthermore, in contrast to the model of McCann *et al*. [4], we did not find that connectivity caused nutrients or phytoplankton biomass to become highly concentrated in the downstream ponds. We expected lower nutrient concentrations and phytoplankton densities in downstream ponds based on dilution, which happens when a fraction of water is moved from a concentrated upstream pond to a less concentrated downstream pond. Indeed, nutrient levels remained higher in the upstream ponds throughout the entire experiment (the upstream pond is where nutrients and lake water, in the case of connected ponds, were supplied).

Because we did not have a strong nutrient pulse across all ecosystems in our experiment, nor did we cease nutrient enrichment at any point during the experiment, we did not observe this gradual washing out of nutrients. By abstracting the movement of water, McCann *et al*. [4] were able to demonstrate the accumulation of nutrients in a ‘terminal’ downstream node, where all nutrients and organisms end up. We suggest that a more explicit model of aquatic connectivity may facilitate the creation of an experimental design that would allow testing the hypothesis of nutrient accumulation in a terminal node.

Although we did not observe nutrient accumulation in the downstream node, connectivity did promote an increase in biomass beyond what might be expected from the movement and dilution of experimentally added nutrients alone. Indeed, in meta-ecosystems where nutrients were added, connectivity had either a positive effect in downstream ponds and a positive or no effect in upstream ponds. This positive effect of connectivity on meta-ecosystem biomass is expected from the spatial insurance effect [12]. In contrast, a microcosm meta-ecosystem experiment showed that resource subsidies to aquatic patches generate weaker increases in heterotrophic protist density when the patch is connected than when it is isolated [35]. Indeed, our results indicate that connectivity had a positive effect on phytoplankton biomass in the upstream patch. One possible explanation is that a decreased abundance of consumers in more highly connected ponds could be responsible for greater phytoplankton biomass. However, this is not supported by our results, which indicate that zooplankton densities were overall very low, less than 0.05 mg ml^−1^ in the most nutrient-rich ponds by the second half of the experiment (figure 5). A more likely explanation is that a nutrient other than the supplied nitrogen and phosphorus became limiting in the nutrient-rich ponds, and the input of mesotrophic lake water to the upstream pond provided a source of this limiting nutrient. This idea is supported by the excess of dissolved phosphorus in upstream ponds, especially in less connected ones, when compared to concentrations in downstream ponds (figure 1). This is also supported by the conceptual model of Olson & Jones [5], which suggests that high water residence times (i.e. low connectivity) can lead to a negative relationship between chlorophyll *a* and total phosphorus concentrations due to limitation by light or another limiting resource. An alternative (though not mutually exclusive) explanation is that connectivity provided increased levels of phytoplankton biodiversity in both patches of the meta-ecosystem, which theory suggests should lead to increased ecosystem functioning, such as biomass productivity [11,12,36]. Indeed, a meta-analysis which includes 44 studies of lake communities reports a mean 46% (95% confidence interval: 29–62%) increase in productivity when comparing the most diverse culture to the average productivity of each species in monoculture [37]. Although we lack sufficient taxonomic data at this time to evaluate this possibility, future work will test this hypothesis using phytoplankton and zooplankton community data collected using eRNA metabarcoding.

The spatial insurance hypothesis states that low to intermediate rates of species dispersal (0.01 to 1% of biomass per dispersal event) between patches in a fluctuating environment can promote asynchronous biomass dynamics among heterogeneous patches [11,12,18]. For example, Thompson *et al*. [38] found that intermediate rates of dispersal can generate asynchronous dynamics in pond communities, though this effect was eroded by a temperature warming treatment. In contrast, we found that connectivity only promoted synchrony, a discrepancy which may be due to multiple factors. First, because rates of dispersal were high (10–40% per week in our experiment), which is known to induce a loss of diversity and synchronize dynamics in models of homogeneous metacommunities. Second, unlike in the model of [11], which assumed persistent environmental heterogeneity (out of phase or random phase cycles of environmental variation) across communities, our study lacked this environmental variation as all the ponds were filled with water from the same source, and all experienced the same weather conditions during the experiment. Third, the relatively small volumes (1000 l) of the ponds led to warmer water during the heat waves of the summer and diel variation in air temperatures, potentially causing synchrony in growing conditions and mortality events across ponds (as in [38]; electronic supplementary material, figure S11). Fourth, our experiment featured a directional flux of nutrients along with dispersal, which further contributed to the environmental synchrony across connected ponds. As a result, flow from an upstream eutrophic pond was unlikely to generate and maintain asynchronous dynamics, at least not in a small environmentally homogenous two-pond meta-ecosystem. New theoretical predictions for spatial insurance effects in linear or dendritic metacommunities would be a valuable guide for future experiments.

Since we did not observe any oscillatory or asynchronous biomass dynamics, it is not surprising that the effect of treatments on temporal stability was limited to the exponential growth phase induced by nutrient enrichment. However, whereas the CV may be effective in measuring variance-driven instability, such as predator–prey cycles, McCann *et al*. [4] suggests that mean-driven, or ‘structural loss of stability’ is more common in natural systems. This type of loss of stability implies a reduction in species diversity within communities. Indeed, we found that zooplankton biomass and diversity exhibited a negative response to nutrient enrichment and a positive response to intermediate levels of connectivity (figures 5 and 6). We propose that high phytoplankton biomass in the most nutrient-rich ponds led to high rates of primary production, depletion of dissolved carbon dioxide (no data), high pH (electronic supplementary material, figure S11) and increased zooplankton mortality. Furthermore, connectivity played a role in the rapid shift from low to high phytoplankton biomass states of meta-ecosystems, particularly in the downstream ponds.

A future experiment will involve a larger number of connected ponds, since network size is known to mediate stability [15], and the control of environmental heterogeneity among the ponds [11,18]. Also, a separate control of nutrient fluxes and rates of organismal dispersal could allow for better comparisons with other meta-ecosystem-oriented studies [23,35]. Finally, here, we selected to approximate a continuous connectivity with episodic, weekly transfers. However, different nutrient pulse and connectivity scenarios could produce different results. For example, a review of extreme weather events suggests that rainfall causes a flushing out of phytoplankton biomass [39]. In contrast, we did not find any evidence of flushing. Indeed, Olson & Jones [5] predicted a negative effect of flushing at a mean water residence time of under 10 days, whereas this value was approximately 2.5 weeks for our most highly connected ponds. The frequency of nutrient enrichment could also affect the temporal stability of phytoplankton, although it is not exactly clear how, since the few experiments comparing press and pulse perturbations report opposing results [40,41]. Additionally, consumers may play an important role in the phytoplankton response to perturbations [42]. As a result, future experiments could compare the effect of nutrient pulses, such as from extreme weather events, to a more continuous flow reminiscent of a continuous supply from an already eutrophic system.

In summary, we tested the hypothesis that connectivity and nutrient enrichment will have interactive effects on the productivity and stability of simple meta-ecosystems. We found that connectivity enhanced the effect of nutrient enrichment on phytoplankton biomass across the meta-ecosystem, beyond what might be expected from nutrient enrichment alone. That is, more connected meta-ecosystems produced more biomass per unit enrichment than less connected meta-ecosystems. We also found that connectivity, especially paired with nutrient enrichment, generated synchronous dynamics of phytoplankton biomass between connected ponds, suggesting that productivity would be more homogeneous across a connected landscape. Synchronous dynamics can also be a prerequisite for destabilizing temporal dynamics, but we found that in most ponds, phytoplankton biomass reached a new steady state within two weeks of the start of the experiment. Perhaps due to a lack of environmental heterogeneity, we did not find any evidence of spatial insurance at intermediate levels of connectivity in terms of temporal stability of phytoplankton biomass. Instead, our results suggest that linear connectivity is more likely to exacerbate eutrophication than stabilize temporal fluctuations across the meta-ecosystem. Furthermore, we found a negative effect of nutrient enrichment on zooplankton biomass and diversity, supporting the idea of McCann *et al.* [4] that ‘mean-driven’, instead of temporal instability, is a more likely outcome of eutrophication in natural aquatic ecosystems. At low levels of nutrient enrichment, however, zooplankton biomass and diversity were enhanced at intermediate levels of connectivity. In short, we conclude that in the context of eutrophication, increasing connectivity between aquatic ecosystems is likely to exacerbate the effects of eutrophication, such as the buildup of phytoplankton biomass and the loss of zooplankton biomass and diversity across all connected ecosystems.

## Data Availability

Data and code are available on Dryad [43]. Supplementary material is available online [44].
